# Orthosis reduces breast pain and mechanical forces through natural and augmented breast tissue in women lying prone

**DOI:** 10.1186/2045-709X-22-2

**Published:** 2014-01-13

**Authors:** Karin Ried, Simon Armstrong, Avni Sali, Patrick McLaughlin

**Affiliations:** 1 National Institute of Integrative Medicine, Melbourne VIC 3122, Australia; 2 College of Health and Biomedicine, Victoria University, Melbourne VIC 3001, Australia

**Keywords:** Breast implant, Breast augmentation, Breast reconstruction, Implant rupture, Lactation, Orthosis, Pain, Mechanical forces, Pressure, Displacement

## Abstract

**Background:**

Breast implant displacement or rupture can cause aesthetic problems and serious medical complications. Activities with prone positioning and loading of the anterior chest wall, such as massage, chiropractic or osteopathic therapies may increase the risk of implant failure and can also cause discomfort in women with natural breast tissue. Here we test the effectiveness of a newly developed orthosis on pain, mechanical pressure and displacement of breast tissue in women with cosmetic augmentation, post-mastectomy reconstruction, lactating or natural breast tissue.

**Methods:**

Thirty-two females volunteers, aged 25–56 years with augmented, reconstructed, natural or lactating breast tissue and cup sizes B-F, participated in this open-label clinical trial. We measured pain perception, peak pressure, maximum force, and breast tissue displacement using different sizes of the orthosis compared to no orthosis. Different densities of the orthosis were also tested in a subgroup of women (n = 7). Pain perception was rated using a validated 11-point visual-analogue scale. Peak pressure and maximum force were assessed using a bilateral set of capacitance-pliance® sensor strips whilst participants were load bearing in a prone position, and breast displacement was measured by magnetic-resonance-imaging.

**Results:**

The orthosis significantly reduced pain, breast displacement and mechanical pressures in women with natural and augmented breast tissue in prone position. Greater relief of pain and greater reduction in mechanical forces were found with increased size and density of the orthosis. Use of the orthosis improved overall comfort by 64-100%, lowered peak pressure by up to 85% and maximum force by up to 96%. Medio-lateral displacement of breast tissue was reduced by 16%, resulting in a 51% desirable increase of breast tissue height.

**Conclusion:**

Our study demonstrated that the newly developed orthosis significantly reduced pain, mechanical pressure and breast tissue displacement in women with augmented and natural breast tissue when lying prone. Our findings are of clinical significance, potentially reducing the risk of complication from prone activities in women with breast augmentation or reconstruction, as well as improving comfort whilst undergoing prone procedures.

**Trial registration:**

Australia and New Zealand Clinical Trials Register, ACTRN12613000541707.

## Background

Clinical and ongoing management following cancer mastectomy and reconstruction, cosmetic augmentation or normal variations in breast tissue structures can present a significant challenge. Some women experience discomfort and potential trauma in clinical settings, when participating in activities that load the anterior chest wall, such as massage, chiropractic or osteopathic therapies. Clinicians need to be aware of altered breast structures and considerations must be given to appropriately manage and accommodate these patients. A new orthosis has been developed to allow women with natural or augmented breast tissue to increase comfort and protection from mechanical forces in the clinical setting or in some recreational activities when lying prone [1].

Breast augmentation has been controversial, especially in light of the French Poly Implant Prothèse (PIP), leading to increased problems and causing anxiety in the perception of this medical field [2,3]. Recent improvements in implant technology and surgical approaches have assisted in improving the outcomes for many individuals. Implants are not for life and will require surgical revision and management [4,5]. Implant rupture rates vary and may be up to 57% at 11 years, with PIP implants rupturing 2–6 times more often than other implants [6]. The biodurability of the implant is of significant concern to patients [7]. In addition, recent court cases on breast implant damage due to manipulation by therapists were decided in favour of the patient [8].

Insertion of a foreign body produces a normal immune response of encapsulation of the object [9]. The body then requires time for the implant to ‘settle’, which can be an ongoing process when complicated by reconstructive, post mastectomy or radiation treatment [10].

Positioning or undertaking activities where loading through the tissues of the anterior chest wall occurs may alter the state of function of breast tissue and/or implant material. This can be highlighted by imaging techniques. Capsular contracture is the most common complication of breast augmentation and can result in collagen fibres of the implant pocket impacting the breast or implant material, which can lead to hardening and resultant asymmetry [11].

Our study investigated mechanical forces, displacement and subjective pain levels of women with natural and surgically altered breasts in the prone position, with and without an orthosis, of different sizes and densities.

## Methods

### Study design and participants

Our study comprised of a non-randomised open-label clinical trial of 32 female volunteers, aged 25–56 years, with breast cup sizes ranging from B-F. About two thirds had augmented breast tissues, most for aesthetic purposes (n = 18), and a few had implants after mastectomy (n = 3). About one third of women in the trial had natural breast tissue, with normal (n = 7), lactating (n = 2), after lumpectomy (n = 1), or reconstruction by tram-flap (n = 1). All but one women in the augmented group had bilateral, complete, silicone implants, with no capsular contracture, which were performed by infra-mammary incision, either with submuscular positioning (n = 13) or subglandular positioning (n = 4). One women in the augmented group had a one-sided reconstruction after mastectomy (n = 1) (Table 1).

**Table 1:** **Baseline characteristics**, **N** = **32**

**Characteristics**	**Mean**	**SD**	**Range**
*Age (yrs)*	36.7	9.7	25-56
*Height (cm)*	166.7	7.3	150-179
*Weight (kg)*	64.2	7.1	51-80
*BMI (kg/m*^*2*^*)*	23.1	2.7	18-31.6
*Cup-size*	4	1.9	B-F
	**N**		
*B*	1		
*C; CC*	8;5		
*D; DD*	8;2		
*E; EE*	4;2		
*F*	2		
*Type*			
*A) Augmentation*^*1*^*:*		**Details**	
Primary:	18	For aesthetic purposes (5.2 ± 3.0 yrs)	
Reconstruction:	3	After mastectomy (4.3 ± 1.5 yrs)	
*B) Natural:*			
Normal	7		
Lactating	2	2-3 months	
Lumptectomy	1	3 months since operation	
Tram flap	1	12 months since operation	

^1^All silicone implants, bilateral, complete, no capsular contracture by infra-mammary incision, with submuscular positioning (n = 13), subglandular (n = 4), or reconstruction (n = 1).

*Abbreviations: mth* month, *n* number, *SD* standard deviation, *yrs* year.

All measurements were taken with the participant lying prone for up to 1 hour during a single day of testing. Magnetic Resonance Imaging (MRI) was performed at Medical Imaging Australia (MIA Victoria) in East Melbourne (45 min), and mechanical forces (peak pressure and maximum pressure were tested at the Victoria University biomechanics lab (15 min). Transport was provided between the testing labs. Pain perception, tissue displacement measured by MRI (4 MRIs per participant) and mechanical forces during prone loading through the anterior chest and breast tissue without and with different sizes (sizes 1–3) of the orthosis were assessed in all participants. In addition, mechanical forces were tested with different densities (soft, medium, firm) of the orthosis in a subgroup of women.

The study was approved by the Human Research Ethics Committee at the National Institute of Integrative Medicine, Melbourne, Australia.

### The orthosis

Made from medical grade thermoplastic elastomer, the orthosis absorbs, deflects and displaces load, protecting the breast structure from trauma and reduces loading of adjacent tissues. The orthosis demonstrates unique elasticity, durability and elastic strain properties, whilst being capable of long term deformation under load. The orthosis is sterilisable in autoclaves and washable with isopropyl, making it suitable for clinical settings and multi-use environments.

**Figure 1:**
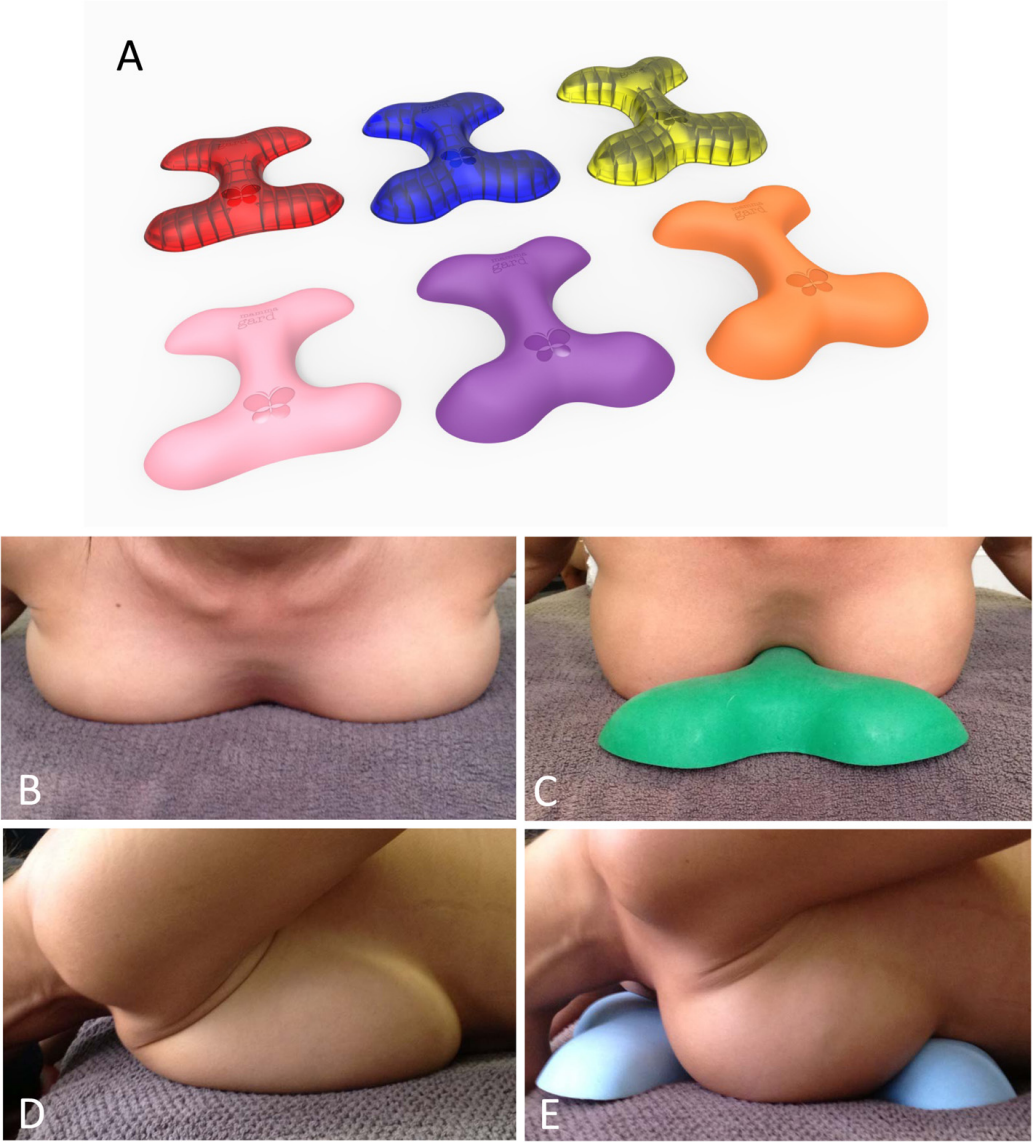
Orthosis in different sizes and densities, the transparent models illustrate the orthosis’ ribbing for adjustment of firmness (A); Participant positioning on the orthosis in cephalic view (B), side view (C), lateral view without the orthosis (D) and with the orthosis (E).

The orthosis device loads the sternum, upper and lower rib cage and upper abdomen (Figure 1). Incremental increases in height, depth and width between the sizes of the orthosis provides allowances for different breast sizes and other structural variations and individual preferences. Size-3 of the orthosis is about 60% higher, 9% wider and 6% longer than the smallest size (size-1) (approximate dimensions of size-1 orthosis: W × L × H = 230 × 260 × 35 mm^3^).

### Pain

Comfort/pain levels were assessed without and with each of 3 sizes of the orthosis. Pain was assessed using a validated 11-point visual-analogue pain rating scale, ranging from 0=’no pain’ to 10=’worst possible pain’ [12,13].

### Breast displacement and deformation, mechanical force and peak pressure

To measure breast tissue displacement and deformation we used Magnetic Resonance Imaging (MRI) in antero-posterior (AP) and medio-lateral (ML) planes. MRIs were performed to show segmental transverse and para-sagittal mid-breast views, providing linear measurements in millimetres (mm) of breast tissue displacement and compression (Siemens 1.5 T Magnetom Espree) [14].

Following MRI scans, mechanical force assessments were taken with subjects lying prone wearing a 15 kilogram load-vest, simulating therapeutic massage and manipulation loading. Bilateral capacitance pliance® sensor strips were used as a means of measuring force (Newton, N) and pressure (kilo pascal kPa) from the breast tissue. Two 8 × 25 cm sensor strips with sensor resolution of 1 sensor/cm^2^ and a total measurement area of 400 cm^2^, sensitive to 4 kPa at a sample rate of 50 Hz, were placed onto a standard treatment table under each of the participants’ breasts. Sensors were aligned to a standardised scale assuring comparable positioning for all participants.

### Sample size and analysis

A sample size of 30 was calculated to detect a difference of 5 ± 2.3 kPa of peak pressure or 25 ± 13 N of maximum force with the orthosis compared to no orthosis with a power of 80% and 95% confidence [1].

Statistical analyses were completed with SPSS Statistics program version 21.0 [15]. Statistical significance was set at p < 0.05. All data were analysed descriptively.

Perceived levels of pain using different sizes of the firm density orthosis compared to no orthosis were assessed by ANOVA repeated measures (General Linear Model univariate repeated measures) with Bonferroni adjustment. The ratio between any pain and no pain was tested by chi-square. Analyses were completed for all participants and by subgroups (primary augmentation, reconstruction, natural, lactating).

Maximum force and peak pressure for each breast side were compared between different sizes of the orthosis and no orthosis using the ANOVA Repeated Measures model for all participants and by subgroups. In addition, different densities of the orthosis as well as sizes compared to no orthosis were tested in a subset of patients (total n = 6; augmented n = 5; natural n = 1).

## Results

### Pain

All women reported a significant reduction of pain with the orthosis compared to no orthosis when lying prone (p < 0.0001; Table 2). The larger size orthosis provided generally greater relief than the smaller size, overall reducing pain sensation by 64-100%. In our patient group, the size-3 orthosis provided complete relief and greatest comfort (p < 0.0001). Average pain perception with no orthosis was slightly higher in augmented patients compared with natural non-lactating patients. The patient with the tram-flap reconstruction did not have any sensation in the reconstructed breast tissue at all and therefore could not report any pain. Generally, smaller breast cup sizes achieved pain reductions with the smaller devices, compared to the larger breast cup sizes which required the larger devices to achieve adequate pain reduction. Comfort data was not captured with the different densities of the orthosis in the subgroup.

**Table 2:** Effect of orthosis on pain scores

	**Pain score**		**Orthosis vs no orthosis**	**Any pain vs no pain**
**All participants (n = 31)**^**1**^	**Mean (SD)**	**% change vs no orthosis**	**ANOVA; p-value**	**Chi-square; p-value**
	
No orthosis	4.8 (1.9)			
Size-1	1.4 (1.0)	-71	<0.0001	0.02
Size-2	0.2 (0.4)	-96	<0.0001	<0.0001
Size-3	0	-100	<0.0001	<0.0001
***Primary augmentation (n = 18)***				
No orthosis	4.7 (1.8)			
Size-1	1.7 (1.0)	-64	<0.0001	
Size-2	0.2 (0.4)	-96	<0.0001	
Size-3	0	-100	<0.0001	
***Reconstruction (n = 3)***				
No orthosis	4.3 (0.6)		0.01	
Size-1	1.0 (0)	-77		
Size-1	0.3 (0.6)	-93	0.006	
Size-3	0	-100		
***Natural (n = 7)***				
No orthosis	4.0 (1.6)			
Size-1	1.3 (1.1)	-68	<0.0001	
Size-2	0.4 (0.5)	-90	<0.0001	
Size-3	0	-100	<0.0001	
***Lactating (n = 2)***				
No orthosis/Size-1/2/3	8/1.5/0/0	-81,-100		
***Lumptectomy (n = 1)***	9/0/0/0	-100		
***TramFlap (n = 1)***^***1***^	0/0/0/0	no pain sensation		

Participants experienced significantly less pain with the orthosis compared to no orthosis.

^1^The patient with the tram-flap reconstruction did not have any sensation in the breast, therefore did not experience any pain. This patient was excluded from the analysis of all patients.

Ns, not significant; vs, versus.

### Mechanical forces

**Figure 2:**
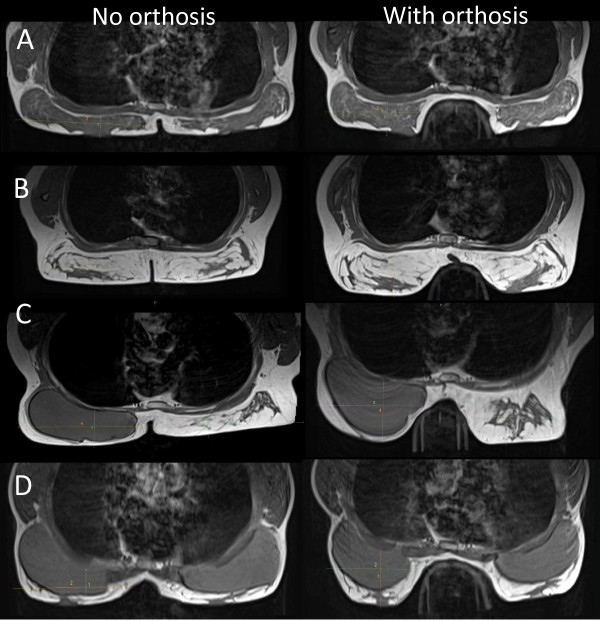
**Assessment of breast displacement.** MRI transverse view of natural breast C-cup **(A)**; lactating breast EE-cup **(B)**; unilateral reconstruction C-cup **(C)**; primary augmentation DD-cup **(D)**; all without (left panel) and with orthosis (right panel).

**Figure 3:**
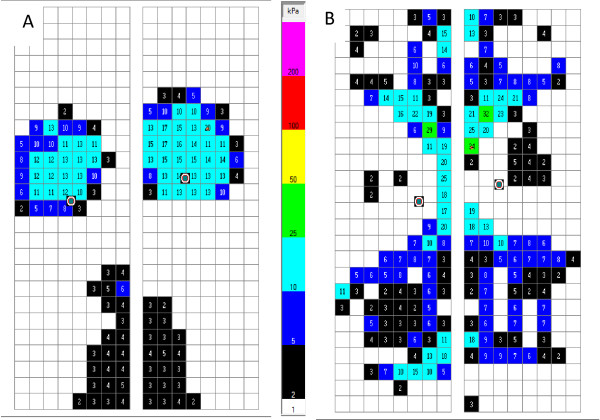
**Assessment of mechanical forces.** Plane view of loading through breast tissue without **(A)** and with the orthosis **(B)**, where pressure (kPa) is distributed along the orthosis.

Peak pressure, maximum force and displacement of breast tissue were significantly reduced for all patients using the orthosis compared with no orthosis (p < 0.001; Table 3A; Figures 2 and 3). The larger size orthosis generally reduced mechanical forces more than the smaller size orthosis (Reduction of forces: Size-3 > Size-2 > Size-1 > no orthosis). The peak pressure reductions observed using the orthosis compared with no orthosis were between 58% and 85% for both breasts for all patients, and maximum force reduction ranged from 73-96%. Breast tissue displacement in the medio-lateral plane was reduced by 14-16%, resulting in a desired increase of 15-51% in the antero-posterior plane.

**Table 3:** Effect of orthosis on mechanical forces

**Outcome**	**Participants**	**Side**	**No orthosis**	**Size-1**	**Size-2**	**Size-3**	**Contrast**	**Change vs no orthosis**	**ANOVA repeated measures**
**A)**	**All (n = 32)**		**Mean (SD)**	**Mean (SD)**	**Mean (SD)**	**Mean (SD)**		%	**p**-**value**
**Peak pressure (kPa)**		left	14.9 (7.4)	6.0 (3.6)	4.5 (3.0)	2.3 (2.1)	S0 vs S1:	-60	<0.001
							S2:	-70	
							S3:	-85	
		right	15.8 (8.4)	6.6 (4.6)	4.8 (3.7)	2.3 (2.1)	S0 vs S1:	-58	<0.001
							S2:	-70	
							S3:	-85	
**Max force (N)**		left	55.0 (20.4)	14.7 (10.2)	6.9 (5.9)	2.3 (2.6)	S0 vs S1:	-73	<0.001
							S2	-87	
							:S3:	-96	
		right	58.8 (18.7)	15.1 (10.0)	7.3 (6.3)	2.7 (3.0)	S0 vs S1:	-74	<0.001
							S2:	-88	
							S3:	-95	
**Displacement (cm)**	**All (n = 32)**	ML	14.6 (1.9)	12.5 (1.8)	12.1 (1.7)	12.2 (1.4)	S0 vs S1:	-14	<0.001
		right					S2:	-17	
							S3:	-16	
		AP	4.1 (1.3)	4.7 (1.3)	5.4 (1.1)	6.2 (1.1)	S0 vs S1:	15	<0.001
		right					S2:	32	
							S3:	51	
**B)**	**Subgroups**								
**Peak pressure (kPa)**	**Without implant (n = 11)**	left	12.6 (4.6)	4.2 (3.7)	3.2 (3.2)	2.1 (2.5)	S0 vs S1:	-67	0.001
							S2:	-75	
							S3:	-83	
		right	13.2 (4.9)	4.8 (3.9)	3.3 (3.3)	2.0 (2.4)	S0 vs S1:	-64	0.006
							S2:	-75	0.001
							S3:	-85	0.001
	**With implant (n = 20-21)**^**1**^	left	16.1 (8.6)	6.6 (2.8)	5.1 (2.7)	2.5 (1.9)	S0 vs S1:	-59	0.002
							S2:	-68	<0.001
							S3:	-84	<0.001
		right	17.1 (9.5)	7.6 (4.8)	5.6 (3.7)	2.5 (2.0)	S0 vs S1:	-56	0.001
							S2:	-67	<0.001
							S3:	-85	<0.001
**Max force (N)**	**Without implant (n = 11)**	left	48.8 (14.4)	10.1 (11.9)	4.6 (6.5)	1.9 (3.3)	S0 vs S1:	-79	<0.001
							S2:	-91	
							S3:	-96	
		right	61.4 (20.9)	18.1 (8.2)	8.9 (6.0)	3.0 (2.7)	S0 vs S1:	-71	<0.001
							S2:	-86	
							S3:	-95	
	**With implant (n = 20**-**21)**^**1**^	left	60.4 (20.7)	17.8 (8.0)	8.4 (5.1)	2.7 (2.2)	S0 vs S1:	-71	<0.001
							S2:	-86	
							S3:	-96	
		right	53.6 (12.8)	9.4 (10.6)	4.3 (6.0)	2.0 (3.7)	S0 vs S1:	-82	<0.001
							S2:	-92	
							S3:	-96	
**Displacement (cm)**	**Without implant (n = 11)**	ML	14.5 (1.8)	12.3 (2.5)	11.4 (2.4)	13.2 (1.6)	S0 vs S1:	-15	0.023
		right					S2:	-21	0.019
							S3:	-9	0.023
		AP	3.1 (1.4)	4.1 (1.6)	5.0 (1.2)	6.4 (1.1)	S0 vs S1:	32	0.023
		right					S2:	61	0.051
							S3:	106	0.002
	**With implant (n = 21)**	ML	14.6 (1.9)	12.7 (1.0)	12.4 (1.1)	11.9 (1.3)	S0 vs S1:	-13	0.001
		right					S2:	-15	0.002
							S3:	-18	<0.001
		AP	4.6 (0.9)	5.2 (0.9)	5.6 (1.1)	6.1 (1.1)	S0 vs S1:	13	0.002
		right					S2:	22	<0.001
							S3:	33	<0.001
**Peak pressure (kPa)**	**Natural (n = 7)**	left	13.0 (2.8)	4.3 (4.1)	3.4 (3.4)	2.4 (2.4)	S0 vs S1:	-67	0.014
							S2:	-74	0.006
							S3:	-82	0.002
		right	13.3 (3.6)	5.1 (4.4)	3.4 (3.4)	2.4 (2.4)	S0 vs S1:	-62	0.084
							S2:	-74	0.018
							S3:	-82	0.007
	**Lactating (n = 2)**	left	15.0 (11.3)	7.0 (0)	5.5 (2.1)	3.0 (4.2)	S0 vs S1:	-53	na
							S2:	-63	
							S3:	-80	
		right	16.0 (11.3)	7.0 (0)	6.0 (1.4)	2.5 (3.5)	S0 vs S1:	-56	na
							S2:	-63	
							S3:	-84	
	**Primary augmentation (n = 18)**	left	16.6 (8.9)	6.7 (2.9)	5.1 (2.9)	2.4 (2.0)	S0 vs S1:	-60	0.003
							S2:	-69	0.001
							S3:	-86	<0.001
		right	16.6 (11.3)	7.0 (0)	6.0 (1.4)	2.5 (3.5)	S0 vs S1:	-58	0.003
							S2:	-64	0.001
							S3:	-85	<0.001
	**Reconstruction (n = 2-3)**^**1**^	left	11.5 (0.7)	5.5 (0.7)	5.0 (0.7)	3.0 (0)	S0 vs S1:	-52	na
		(n = 2)					S2:	-57	
							S3:	-74	
		right	20.3 (14.4)	12.7 (10.7)	9.0 (7.8)	2.0 (1.7)	S0 vs S1:	-37	na
		(n = 3)					S2:	-56	
							S3:	-90	
	**Lumpectomy (n = 1)**	left	8	0	0	0	S0 vs all:	-100	na
		right	10	0	0	0	S0 vs all:	-100	
	**Tram flap (n = 1)**	left	10	2	0	0	S0 vs all:	-80,-100	na
		right	10	3	0	0	S0 vs all:	-70,-100	
**Max force (N)**	**Natural (n = 7)**	left	52.3 (13.9)	11.6 (13.1)	5.0 (7.0)	2.1 (3.7)	S0 vs S1:	-78	0.005
							S2:	-90	0.001
							S3:	-96	0.001
		right	51.4 (12.0)	10.1 (11.4)	4.0 (5.6)	2.1 (4.1)	S0 vs S1:	-80	0.005
							S2:	-92	0.001
							S3:	-96	<0.001
	**Lactating (n = 2)**	left	45.5 (17.7)	14.5 (12.0)	8.0 (8.5)	3.0 (4.2)	S0 vs S1:	-68	na
							S2:	-82	
							S3:	-93	
		right	47.0 (15.6)	15.0 (11.3)	9.5 (9.2)	3.5 (4.9)	S0 vs S1:	-68	na
							S2:	-80	
							S3:	-93	
	**Primary augmentation (n = 18)**	left	59.0 (21.2)	17.5 (8.4)	8.2 (5.2)	2.6 (2.3)	S0 vs S1:	-70	<0.001
							S2:	-86	
							S3:	-96	
		right	60.8 (21.1)	18.1 (8.7)	9.2 (6.4)	2.9 (2.7)	S0 vs S1:	-70	<0.001
							S2:	-85	
							S3:	-95	
	**Reconstruction (n = 2-3)**^**1**^	left	72.5 (13.4)	20.5 (5.0)	10.5 (5.0)	3.0 (1.4)	S0 vs S1:	-72	na
							S2:	-86	
							S3:	-96	
		right	65.0 (23.6)	18.3 (4.7)	7.3 (2.5)	4.0 (2.6)	S0 vs S1:	-72	na
							S2:	-89	
							S3:	-94	
	**Lumpecto**	left	27	0	0	0	S0 vs all:	-100	na
	**my (n = 1)**	right	70	0	0	0	S0 vs all:	-100	
	**Tram flap**	left	53	1	0	0	S0 vs all:	-98,-100	na
	**(n = 1)**	right	66	2	0	0	S0 vs all:	-97,-100	

A) All participants; B) Subgroups. All orthoses tested were of firm density.^1^ One participant had a one sided (right side) implant after mastectomy. Subgroup analysis includes the participant’s measures for the right side only.

AP, antero-posterior; kPa, kilopascal; ML, medio-lateral; N, Newton; n, number; na, not applicable; S0, no orthosis; S1, size-1 orthosis; S2, size-2 orthosis; S3, size-3 orthosis; vs, versus

Table 3B summarises subgroup analysis results on peak pressure, maximum force and displacement by patients with or without implants, and by natural, lactating, primary augmentation or reconstruction. Reductions in peak pressure and maximum forces were similar in women with or without implants using the orthoses, while reduction of displacement was greater in women without implants. The effect of the orthosis on mechanical forces was comparable in all subgroups.

**Figure 4:**
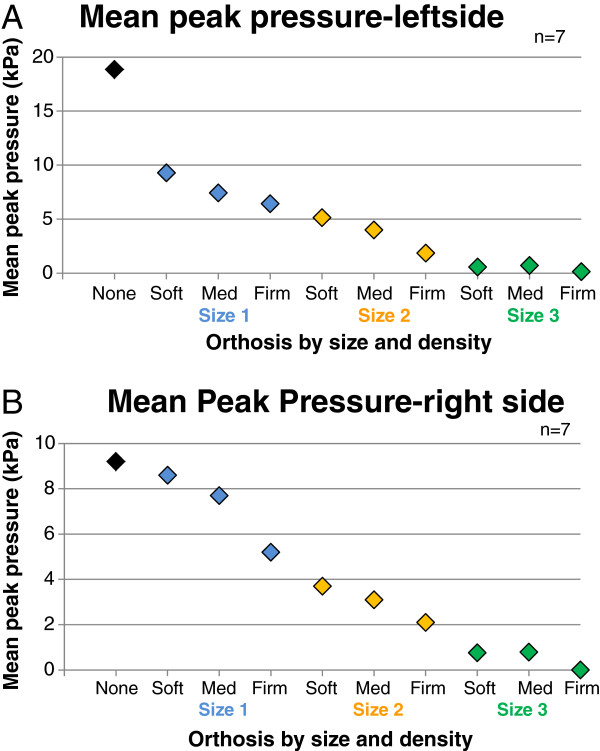
Mean peak pressure by orthosis size (sizes 1–3) and density (soft, medium, firm) on the left (A) or right breast side (B) in a subgroup of participants compared with no orthosis (black diamond).

The effect of difference densities of the orthosis on peak pressure was tested in a subgroup of women (n = 7). Mean peak pressure dropped with increasing size and density of the orthosis, with firmer and larger orthosis providing greater decrease in mechanical forces (Figure 4). Almost no peak pressure was observed with the firmer variant of the size-3 orthosis in this group of women.

**Figure 5:**
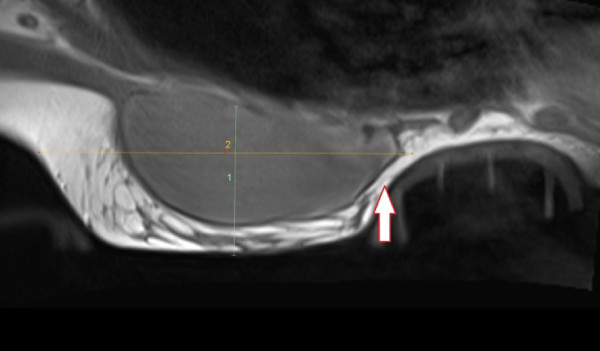
**Sagittal MRI view mid-breast of an E-cup primary augmented individual in a size-1 device.** The arrow points to the implant loading upon the orthosis, as it is too small. This must be avoided.

Table 4 provides a general guide for matching size and density of the orthosis to breast cup-size based on our findings and on clinical practice. Women with A-C cup-size breasts generally require a size-1 orthosis to provide protection. A size-3 orthosis can be used, although there is no functional gain in this group. Women with D-E cup-size require a size-2 orthosis as a minimum, and breast cup-sizes EE and above must only use a size-3 device or larger. Appropriate sizing of the orthosis is crucial as breast tissue and implant material must not load onto the device but be adjacent to it for correct uses allowing optimal protection (Figure 5).

**Table 4:** Guidelines for size and density choice of orthosis by breast cup-size, as suggested (X), optional (O), and to avoid (white)

	**Orthosis size-1**				**Orthosis size-2**				**Orthosis size-3**			
**Breast cup size**	**SSoft**	**Soft**	**Med**	**Firm**	**SSoft**	**Soft**	**Med**	**Firm**	**SSoft**	**Soft**	**Med**	**Firm**
**A+**	X	X	X	X	O	O	O					
**B+**	X	X	X	X	X	O	O	O				
**C+**	O	O	X	X	X	X	X	X	O	O		
**D+**				X	X	X	X	X	X	O	O	O
**E+**					X	X	X	X	X	X	X	O
**F+**									X	X	X	X
**G+**									X	X	X	X
**H+**										X	X	X

Ssoft, supersoft; Med, medium.

The orthosis of supersoft density was not used in this trial.

## Discussion

Our study demonstrated that the new orthosis significantly reduced pain, breast displacement and mechanical pressures in women with natural and augmented breast tissue when undergoing activities in prone position. Greatest comfort and complete pain relief was observed with the largest size-3 orthosis in our group of women with B-F cup-sizes. The orthosis allowed peak pressure to be lowered by up to 85%, maximum force by up to 96%, and medio-lateral displacement of breast tissue by up to 16%, which in turn resulted in up to 51% increase of breast tissue in the antero-posterior plane.

Natural breast tissue demonstrated a greater proportional protection from displacement and deformation with the orthosis compared to augmented individuals. This can be attributed to the implant’s fixed volume and inability to simulate natural human tissue movement.

The findings of this larger study are in line with our earlier pilot study [1]. Here we provide additional data on bilateral measurements and a variety of densities of the orthosis.

Our findings are clinically important to provide lactating women or women with natural painful breast tissue a safer and more comfortable option when undergoing prone activities, such as massage to relief back pain. Additionally, use of the orthosis in women with augmented breast tissue may reduce the risk of rupture or displacement of implant material during activities involving external pressure and mechanical loading of the breast tissues, often involved in massage, chiropractic, osteopathic, and physical therapy modalities [8].

Stiffness, fluidity, elasticity and density vary between tissue and implant material, causing shear strains parallel to the patient’s plane of contact, and when reaching the limits of elasticity the implant material or human tissue may rupture or tear. Patients who have undergone breast reconstruction using implants after breast cancer surgery are likely to have a higher failure rate than primary augmentation patients, as their residual natural tissues are generally more vulnerable [16]. Furthermore, as implant material has no neural innervation the individual may be unaware of the damage, known as silent rupture. Conversely, when human tissue fails and the implant moves, asymmetry such as a symmastia or ‘uni-breast’ is caused [17].

The newly developed orthosis’ structure allows for specificity in load distribution, isolating pressure tolerant areas and relieving sensitive areas (Figure 1a). Further research is needed to capture the breast pain in relation to device density and duration of prone loading. In our study, patients were most comfortable lying prone on the size-3 firm orthosis during the 1 hour testing sessions. Our subgroup study suggests that the softer density device provides less loading protection, but additional studies are needed to ascertain whether softer varieties provide greater rib cage comfort when used for longer periods. In practice lighter subjects generally prefer the softer density device whilst heavier subjects prefer the firmer density device. Comfort and correct fit are key to the orthosis being superior to current methods of using towels, pillows and bolsters. The orthosis is reliable in its capacity, its use is repeatable and results are reproducible, which is important in litigious environments.

Professional fitting by an orthotist or primary healthcare practitioner is recommended to ensure appropriate use and maximum protection (Additional file 1). It is imperative that the altered and ‘at risk’ breast structures be exposed to minimal loading and therefore must be adjacent to the device. The orthosis should be part of ongoing management when breast tissue has been altered. It is advisable that patient have their own orthosis for use in day to day management.

Future research could test the orthosis in situations other than prone, for example in the upright positioning such as car seat travel to reduce the risk of breast implant rupture by the seat belt during an accident [18]. A light harness or fixation system could hold the orthosis in position against the torso. Further larger long-term studies are needed to determine potential risk reduction in complication rates and breast implant longevity with regular use of the orthosis.

## Conclusion

Our research demonstrates the new orthosis to significantly reduce pain, displacement and mechanical pressure in natural and augmented breast tissue in women undertaking prone activities with symmetrical loading across their back. This has significant implications in both clinical settings, and for general activities of the patient in prone position, e.g. massage, orthopaedic treatment. Our study has contributed to developing guidelines for fitting of optimal size and density of the orthosis to reduce loading and increase comfort, also in relation to breast size, and patient weight. In our study, larger and firmer options of the orthosis showed generally greater effectiveness in our group of women with breast cup-sizes B-F. Clinicians placing patients in prone positions are encouraged to use the orthosis. In patients with breast augmentation or reconstruction, to prevent litigation risks, as resultant breast structures are less deformable and may cause greater discomfort in the prone position. Post-operative fitting following breast surgery of any description is advisable to protect the altered tissues and allow for a more safe and comfortable return to activities of daily living.

## Consent

Written informed consent was obtained from the participants for the publication of this report and any accompanying images.

## Competing interests

PM is consultant to novel.de, but none of the companies and centres accessed for this study were involved in the study design, data collection, analysis and preparation of the manuscript. No external or industry funding was received. The authors declare no conflict of interest.

## Authors’ contributions

All authors contributed to the design and planning of the study. SA and PM conceptualised the study, recruited participants, and collected data. KR analysed data and prepared the manuscript for publication with contributions from co-authors. All authors approved the final version.

## Supplementary Material

Additional file 1 **Professional fitting guidelines of MammaGard orthosis. Figure S1.** The manubrio-sternal joint is lying approximately adjacent to the MammaGard name logo (red circle). The logo is engraved on top of the orthosis (refer to Figure 1A in the main manuscript). **Figure S2.** The xiphoid process is adjacent to the butterfly logo (red circle). The logo is engraved on top of the orthosis (refer to Figure 1A in the main manuscript). Click here for file
