# Supplementary material for: Orthosis reduces breast pain and mechanical forces through natural and augmented breast tissue in women lying prone
Source: Chiropr Man Therap. 2014 Jan 13;22:2. doi: 10.1186/2045-709X-22-2 (PMC3896682; doi:10.1186/2045-709X-22-2)
Supplement: Additional file 1 — Professional fitting guidelines of MammaGard orthosis. Figure S1. The manubrio-sternal joint is lying approximately adjacent to the MammaGard name logo (red circle). The logo is engraved on top of the orthosis (refer to Figure 1A in the main manuscript). Figure S2. The xiphoid process is adjacent to the butterfly logo (red circle). The logo is engraved on top of the orthosis (refer to Figure 1A in the main manuscript). [file 2045-709X-22-2-S1.pdf]

Professional fitting guidelines of MammaGard orthosis

The orthosis is used in load bearing positions, such as when sleeping or when undergoing prone examination and treatment.

Generally, women with A-C cup size breasts use size 1 or size 2 devices, women with D-E cup use a size 2 or 3, and women with EE and above must use only size 3 devices - this is to ensure the patients breasts lay adjacent to the orthotic and not upon it.

Ensure the manubrio-sternal joint is lying approximately adjacent to the MammaGard name logo (Appendix Figure 1) and the xiphoid process is adjacent to the butterfly (Appendix Figure 2).

In general, firmer devices provide a greater protection for all breast cup-sizes, however the softer option may be more appropriate for longer duration low impact loading, whilst firmer devices may be more suitable for shorter duration high impact loading.

Practitioners must always be aware of existing structural variations in the breast tissue and relative contraindications for use. See the sizing chart for assistance (Table 4).

Considerations should be given that loading of the sternum and costal cartilages is a new sensation for the user and a little tolerance may have to be developed over time in some patients. Similar to other orthotic devices it may take a gradual period of time to increase tolerability for prolonged use. Some clients may be able to use the device easily and are comfortable with the firm density, whilst others need time to build resilience to the new loadbearing distribution pattern.

The grade/ firmness of the device is influenced by the individual's weight and the firmness of the surface they are lying upon, as well as their thoracic cage structure. Generally, women under 55 kg in weight use the softest grade of the orthotic and as the weight of the patient increases firmer devices are required. If the loading surface is hard, patients generally prefer a softer device, and if the surface is soft, firmer grades are preferred. Individual preference of the patient will guide choice of density. Mechanical loading and displacement is reduced the greatest the firmer the device. It may take a few attempts to achieve the most desirable fitting for the patient.

Patients may or may not wear a bra when using the orthosis. However, it is advisable for patients not to wear a bulky or wired bra, nor a garmet with buttons or zippers between the orthotic and their thoracic cage/ upper abdomen as this may cause discomfort or skin irritation.
Fitting should be undertaken once all post-operative healing has occurred. Patient comfort is the most important indicator for fitting.

If the patient has brought their own device, the practitioner can make adjustments to reduce a specific area of loadbearing capacity by cutting and therefore weakening the rib(s) for a softer feel.

The patient may use the orthosis for daily activities such as sleeping prone or in the gymnasium or sport environments. Optimum function occurs when on a flat surface with full contact of the orthotic perpendicular to the thoracic cage.

Indications for use
Presence of implant material to breast structures
Altered breast structure (ie. tram flap reconstruction, mastectomy, lumpectomy)
Painful natural breast tissue such as when lactating and hormonal variations

Relative contraindications for use
Low bone density
Recent history of thoracic cage trauma
Poor skin tissue integrity of load bearing regions
Abnormal variants of thoracic cage

Absolute contraindications for use
Pain on use of device
Osteoporosis
Significant structural abnormality of thoracic cage, i.e. pectus recurvatum or funnel chest

Device maintenance
MammaGard orthotics are isopropanol/isopropyl alcohol tolerant, dishwasher safe and steam autoclave temperature resistant to 121 °C.

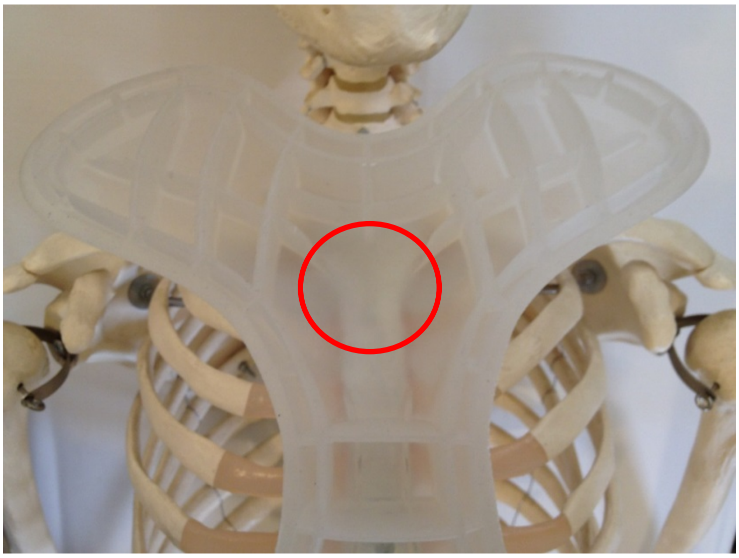

Figure 1
The manubrio-sternal joint is lying approximately adjacent to the MammaGard name logo (red circle). The logo is engraved on top of the orthosis (refer to Figure 1A in the main manuscript).

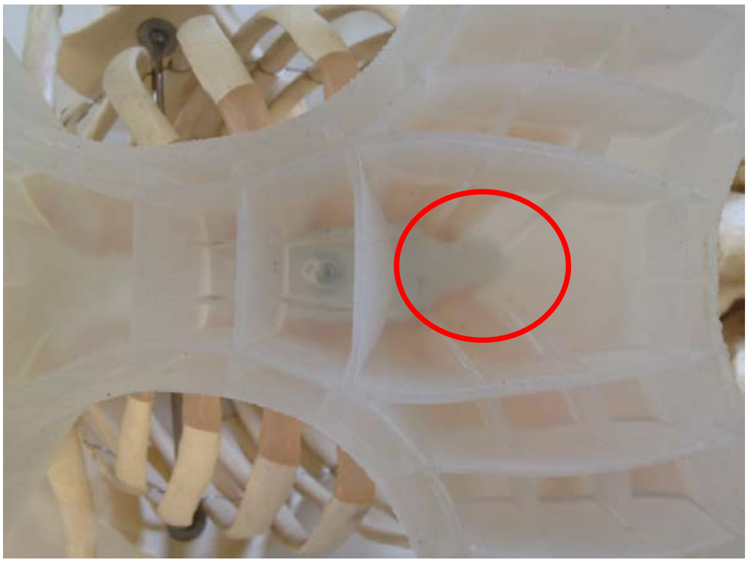

Figure 2
The xiphoid process is adjacent to the butterfly logo (red circle). The logo is engraved on top of the orthosis (refer to Figure 1A in the main manuscript).
